# Extensively Drug-Resistant Tuberculosis,Taiwan

**DOI:** 10.3201/eid1405.071398

**Published:** 2008-05

**Authors:** Ming-Chih Yu, Mei-Hua Wu, Ruwen Jou

**Affiliations:** * Taipei Medical University–Wan Fang Hospital, Taipei, Taiwan, Republic of China; † Centers for Disease Control, Taipei, Taiwan, Republic of China

**Keywords:** Extensively drug-resistant, Mycobacterium tuberculosis, fluoroquinolones, Taiwan, letter

## Abstract

Extensively Drug-Resistant Tuberculosis, Taiwan

**To the Editor:** In Taiwan, the incidence of tuberculosis (TB) was 74.1/100,000 population in 2004 and 72.7/100,000 in 2005; the mortality rate was 4.2/100,000 in 2004 and 4.3/100,000 in 2005 (*1*). Because of these high incidences and the increasing effects of multidrug-resistant TB (MDR TB), i.e., resistant to at least both isoniazid (INH) and rifampin (RIF), the laboratory-based Taiwan Surveillance of Drug Resistance in TB (TSDRTB) program was established in 2003 (*2*). Surveillance demonstrated that combined drug resistance rates were 11.3% (2004) and 10.1% (2005) for INH; 7.5% (2004) and 6.2% (2005) for RIF; 4.3% (2004) and 2.1% (2005) for ethambutol (EMB); 10.6% (2004) and 9.8% (2005) for streptomycin (SM); 20.4% (2004) and 18.1% (2005) for any first-line drug; and 5.3% (2004) and 4.0% (2005) for MDR TB. These resistance rates are higher than those reported by the third TB global drug resistance surveillance. Global surveillance reported median prevalence of combined drug resistance was 6.6% for INH, 2.2% for RIF, 1.3% for EMB, 6.1% for SM, 10.4% for any drug, and 1.7% for MDR TB (*3*).

Extensively drug-resistant TB (XDR TB) was initially defined as an MDR isolate that was resistant to at least 3 of the 6 main classes of second-line drugs: aminoglycosides, polypeptides, fluoroquinolones, thioamides, cycloserine, and para-aminosalicylic acid (*4*). In October 2006, the World Health Organization (WHO) redefined XDR TB as an isolate “resistant to at least INH and RIF (i.e., MDR TB) plus resistant to at least 1 of the fluoroquinolones and 1 of the following 3 injectable drugs: capreomycin, kanamycin, and amikacin” (*5*). Clearly, XDR TB is a global threat and the demands on XDR TB surveillance systems are urgent.

Because no guidelines for drug susceptibility testing of second-line drugs existed in Taiwan before 2007, clinical mycobacteriology laboratories performed drug susceptibility testing of second-line drugs using the agar proportion method by clinicians’ request only. Critical concentrations of second-line drugs for drug susceptibility testing were 2 μg/mL for ofloxacin, 6 μg/mL for kanamycin, 10 μg/mL for ethionamide, and 8 μg/mL for para-aminosalicylate. Of the 215 MDR isolates, 92 (42.8%), 35 (16.3%), 34 (15.8%), and 56 (26.0%) were resistant to fluoroquinolone, kanamycin, ethionamide, and para-aminosalicylate, respectively. Of the 116 MDR isolates tested for susceptibility to second-line drugs in 2004, 10.3% (12/116) were XDR TB; of the 99 MDR isolates tested in 2005, 10.1% (10/99) were XDR TB.

With their broad spectrum antimicrobial activity, fluoroquinolones are widely used for the treatment of bacterial respiratory infections in Taiwan. In addition, fluoroquinolones are the preferred oral agents for treating drug-resistant TB that is known or presumed to be sensitive to this class of drugs, or when first-line agents cannot be used because of intolerance (*6*). In contrast to injectable agents that have a higher incidence of renal and hearing impairment after long-term use, fluoroquinolones have high oral bioavailability, convenient dosing intervals, and a lower incidence of side effects (*7*). Therefore, despite TB treatment recommendations, some clinicians prescribe fluoroquinolones instead of injectable agents. A previous study by Yu et al. (*8*) showed that fluoroquinolone-resistant *Mycobacterium tuberculosis* isolates were rare among patients not previously exposed to fluoroquinolones; however, the increased rate of resistance to fluoroquinolones was observed among patients with MDR TB because of inadequate treatment regimens or poor compliance (*8**,**9*).

In this study, 215 MDR isolates were tested; among these, 42.8% (92/215) were fluoroquinolone-resistant, a much higher percentage than the 10.2% (22/215) that fulfilled the definition of XDR TB. Because the adequate use of fluoroquinolones for TB per WHO and national guidelines (either for intolerance or drug resistance) is important, the use of fluoroquinolones is strictly regulated by the National Health Insurance program. Since 2007, clinicians in Taiwan have been required to apply for these and second-line drugs through the Taiwan Centers for Disease Control (CDC) and to accept professional supervision in their administration. Furthermore, these drugs can only be given under the direct observed treatment program.

An outbreak in rural South Africa highlighted the risk of XDR TB for persons co-infected with HIV (*10*). The current Taiwan TB and HIV Register shows that <1% of TB patients are co-infected with HIV and documents no XDR TB patients who are co-infected with HIV. However, because persons co-infected with HIV and *M. tuberculosis* have the highest rates of progression to active disease, continued monitoring of co-infected patients is essential for control of TB.

The initial purpose of the TSDRTB program was to survey drug resistance of first-line anti-TB drugs in Taiwan. Therefore, our study data are limited; the rate of XDR TB among MDR TB may be an underestimate because we did not have adequate representative cases and methods. The present surveillance system does clearly show the emergence of XDR TB cases in Taiwan, which highlights the need to reinforce diagnosis and treatment strategies recommended by the National TB Control Program. In addition to the established TSDRTB program, Taiwan CDC started an enhanced population-based surveillance of MDR TB/XDR TB in 2007.
